# Broad-spectrum antifungal activity and genome-guided characterization of *Paenibacillus polymyxa* CACC1094 isolated from the bovine rumen

**DOI:** 10.1371/journal.pone.0350885

**Published:** 2026-06-25

**Authors:** Sanghun Lee, Jeongsup Song, Yangseon Kim

**Affiliations:** Department of Research and Development, Center for Industrialization of Agricultural and Livestock Microorganisms, Jeongeup-si, South Korea; Yenepoya University, INDIA

## Abstract

While *Paenibacillus polymyxa* is widely recognized for its biocontrol capabilities, most characterized strains originate from soil or rhizosphere environments, leaving animal-associated populations largely unexplored. In this study, we report the isolation of *P. polymyxa* strain CACC1094 from the bovine rumen and its genome-guided characterization to investigate its biosynthetic potential and antifungal activity. Whole-genome sequencing yielded a complete circular chromosome of 5.55 Mb with a GC content of 45.36%, comprising 5,099 coding sequences, 39 rRNA genes, and 111 tRNA genes. Comparative phylogenomic analysis placed CACC1094 within the *P. polymyxa* species complex, clustering most closely with the rumen-associated strain ND24 (ANI: 98.31%) and strain 188 (ANI: 96.92%), while forming a distinct branch within the species. Genome mining identified 13 biosynthetic gene clusters, including those associated with fusaricidin and tridecaptin biosynthesis, a paenicidin-like lanthipeptide cluster, and a hybrid NRPS–PKS cluster. In dual-culture assays, CACC1094 showed broad *in vitro* antifungal activity against multiple plant-pathogenic fungi and oomycetes, as well as selected fungal and yeast pathogens of clinical and veterinary relevance. LC-QTOF/MS analysis of culture supernatants confirmed the production of fusaricidin A and fusaricidin B, providing direct experimental validation of genome-derived predictions. Additionally, genome annotation revealed a complete CRISPR-Cas adaptive immune system, suggesting an integrated ecological strategy that combines antimicrobial biosynthesis with defense against mobile genetic elements. Together, these findings identify CACC1094 as a rumen-associated *P. polymyxa* strain with broad-spectrum antifungal activity and experimentally validated fusaricidin production, highlighting its potential as a source of antifungal metabolites for agricultural and veterinary applications.

## Introduction

Fungal pathogens continue to threaten global food security and animal health. In agriculture, filamentous fungi such as *Fusarium*, *Botrytis*, and *Colletotrichum*, along with oomycetes like *Pythium*, are responsible for annual yield and quality losses estimated at 10–20% worldwide [1–3]. These infections reduce crop productivity and postharvest value, affecting trade and storage [2], and their prevalence and virulence are further aggravated by climate change [4]. In parallel, opportunistic fungi including *Aspergillus*, *Cryptococcus*, *Candida*, and *Malassezia* cause respiratory, systemic, and dermatological diseases in animals and humans [5–7]. The growing emergence of multidrug-resistant strains, such as azole-resistant *Aspergillus fumigatus* and *Candida auris*, highlights the limitations of current control strategies [8–11]. Conventional management still relies heavily on synthetic fungicides and antifungal drugs; however, their effectiveness is increasingly undermined by resistance development, environmental toxicity, bioaccumulation, and tightening regulatory restrictions [12–14]. These challenges underscore the urgent need for sustainable antifungal alternatives, particularly those based on microbial antagonism and ecosystem-compatible biocontrol approaches.

Among microbial candidates, *Paenibacillus polymyxa* has emerged as a versatile biocontrol agent and plant growth-promoting rhizobacterium (PGPR) [15–17]. This Gram-positive, endospore-forming bacterium suppresses diverse pathogens by producing structurally diverse secondary metabolites synthesized by nonribosomal peptide synthetases (NRPSs) and polyketide synthases (PKSs) [18–20]. Well-characterized compounds include fusaricidins, tridecaptins, and paenicidins, which confer potent antifungal and antibacterial activities [16,21,22]. Genome mining of *P. polymyxa* has also revealed additional PKS-like and hybrid NRPS–PKS clusters whose roles remain poorly understood [18–20]. However, despite extensive studies on soil- and rhizosphere-derived *P. polymyxa* strains, their animal-associated counterparts remain largely unexplored, representing a significant gap in our ecological understanding.

The bovine rumen, a dense anaerobic fermentation environment where soil- and plant-derived bacteria are continuously introduced through feed, may serve as a unique ecological niche that drives novel biosynthetic adaptations. Notably, rumen-derived *Paenibacillus* isolates have been reported, indicating that the rumen can harbor cultivable *Paenibacillus* strains with distinct metabolic traits. For example, *Paenibacillus* strain 79R4 was isolated from the bovine rumen and investigated for enhanced nitrite-reducing capacity in the context of ruminal nitrate/nitrite detoxification and fermentation efficiency [23]. In addition, cellulolytic/lignocellulose-degrading rumen isolates such as *P. polymyxa* ND24 and ND25 have been described, highlighting the rumen as a reservoir of enzyme-rich *Paenibacillus* strains adapted to complex plant biomass [24,25]. Other rumen-origin *Paenibacillus* isolates (e.g., *Paenibacillus barengoltzii* A1_50L2) have also been studied as sources of plant cell wall–degrading enzyme cocktails [26]. Collectively, these studies suggest that rumen-associated *Paenibacillus* lineages may encode niche-adapted functions; yet, their secondary-metabolite biosynthetic repertoires and broad-spectrum antifungal potential remain insufficiently characterized, especially in relation to NRPS/PKS-derived metabolite diversity.

Under such competitive and nutrient-rich conditions, rumen-derived *P. polymyxa* strains may harbor expanded or specialized biosynthetic repertoires, enabling antagonistic activity not only against phytopathogens but also against fungi relevant to veterinary health. Moreover, the acquisition of adaptive defense systems such as CRISPR-Cas could further stabilize biosynthetic gene clusters and support their long-term evolution, enhancing ecological fitness and competitive success in microbially dense environments. Consequently, exploring rumen-derived *P. polymyxa* strains may uncover underappreciated metabolic diversity with potential applications beyond conventional biocontrol.

Here, we report a genome-guided characterization of P. polymyxa strain CACC1094, isolated from the bovine rumen. We hypothesized that a rumen-associated *P. polymyxa* isolate might possess biosynthetic features associated with antifungal activity and therefore represent a potentially valuable microbial resource. To test this hypothesis, we combined genome-based BGC analysis, phylogenomics, and metabolite-level validation using LC-QTOF/MS, and linked these genomic insights to phenotypic antifungal activity. We further examined the coexistence of antimicrobial BGCs and CRISPR-Cas adaptive immunity as a potential integrated ecological strategy, thereby broadening our understanding of the biosynthetic potential of *P. polymyxa* and suggesting that rumen-derived strains may represent underexplored sources of antifungal metabolites.

## Materials and methods

### Bacterial strain isolation and identification

Rumen liquid was collected from a Holstein cow and immediately transferred into a pre-warmed insulated vacuum flask with minimal headspace to maintain anaerobic conditions during transport. All samples were processed within 2 h of collection without prior enrichment. For bacterial isolation, the rumen liquid was serially diluted in sterile saline (0.85% NaCl, w/v) from 10^-1^ to 10^-6^. Aliquots (100 µL) of selected dilutions (10^-2^ to 10^-6^) were spread onto a variety of agar media, including Reinforced Clostridial Medium (RCM), Chopped Meat Medium (CMM), Selenomonas ruminantium medium, Yeast Extract Sodium Lactate (YESL) Medium, MRS, and nutrient agar (NA), to recover a broad range of cultivable bacteria. The plates were incubated under anaerobic conditions at room temperature (22–23°C) for 48 h.

Following incubation, multiple bacterial colonies were recovered across the different media. To maximize phenotypic diversity, colonies displaying distinct morphologies and pigmentation patterns were preferentially selected. When multiple colonies exhibited similar colony morphology, at least three colonies were independently picked to minimize clonal bias. The selected colonies were purified by at least two successive streak-plating steps on fresh agar plates to obtain single-colony isolates.

For taxonomic identification, the 16S rRNA gene was amplified from genomic DNA extracted from the purified isolates using the universal primers 518F (5′-CCAGCAGCCGCGGTAATAC-3′) and 805R (5′-GACTACCAGGGTATCTAATC-3′). The resulting PCR products were sequenced and compared against the National Center for Biotechnology Information (NCBI) database using the BLAST algorithm to assess sequence similarity and assign species identity. Among the recovered isolates, one strain identified as Paenibacillus polymyxa was designated CACC1094 and selected for further phenotypic and genomic characterization.

### Antifungal activity assay

The antifungal activity of *Paenibacillus polymyxa* CACC1094 was evaluated using dual-culture assays against a diverse set of pathogens, including filamentous fungi, yeasts, and oomycetes, that are associated with plant, veterinary, and human diseases. Filamentous fungi and the oomycete were cultured on potato dextrose agar (PDA) at 25°C for 5–7 days. An 8 mm agar plug was cut from the actively growing edge of each colony and placed at the center of a fresh PDA plate. Yeast cells were cultivated in potato dextrose broth (PDB) or Sabouraud dextrose broth (SDB) at 30°C with shaking for 24 h. After adjusting the cell density, 200 μL of the suspension was evenly spread onto PDA or SDA plates, depending on the optimal medium for each species. Four sterile 8 mm paper disks were initially placed equidistantly at the left, right, top, and bottom positions relative to the center of the plate. A 20 μL suspension of *P. polymyxa* CACC1094 was applied to the disks on the left and right sides, while the top and bottom disks were treated with 20 μL of hygromycin B (50 mg/mL) and sterile LB medium, respectively, as positive and negative controls. Plates were incubated at 25°C for filamentous fungi and oomycetes, and at 30°C for yeasts. Antifungal activity was evaluated by visually assessing the formation of clear inhibition zones surrounding the bacterial disks after 5–7 days for filamentous fungi and oomycetes, and after 48 h for yeasts. The size of the inhibition zone was determined by measuring the distance (mm) from the edge of the bacterial disk to the nearest visible fungal or yeast growth front. The fungal pathogens used for the antagonism assay are listed in S1 Table.

### Hybrid whole-genome sequencing

Genomic DNA was extracted from *P. polymyxa* CACC1094 using a commercial bacterial genomic DNA isolation kit (QIAGEN), following the manufacturer’s protocol. Whole-genome sequencing was performed using a hybrid approach combining two platforms: the Illumina NovaSeq 6000 system (paired-end 150 bp) for high-accuracy short reads, and the Oxford Nanopore MinION system for long-read sequencing. Raw sequencing data from both platforms were demultiplexed and subjected to initial quality control. Illumina reads were assessed using FastQC [27], and Nanopore reads were evaluated using NanoPlot [28]. Adapter sequences and low-quality bases were trimmed from Illumina reads using Trimmomatic [29], and Nanopore reads were filtered using NanoFilt [28] to remove reads below the quality threshold. Post-trimming quality was reassessed using FastQC for Illumina and NanoStat for Nanopore datasets. To remove potential contaminant sequences, both Illumina and Nanopore datasets were screened using Kraken2 [30] against a comprehensive microbial reference database. Only high-confidence reads were retained for downstream genome assembly.

### Genome assembly, annotation and bioinformatics analysis

Initial *de novo* genome assembly was conducted using high-quality Illumina reads in Unicycler (v0.4.8) hybrid mode, which integrates both short and long reads [31]. Illumina-based assemblies were polished using Pilon, guided by read mapping with BWA-MEM [32], and long Nanopore reads were then incorporated to bridge contigs and verify genome circularization. This hybrid strategy facilitated the resolution of repeat-rich regions and generated a high-contiguity, gapless genome. Structural and functional annotation of the finalized genome was conducted using the NCBI Prokaryotic Genome Annotation Pipeline (PGAP). Predicted coding sequences (CDSs), rRNAs, and tRNAs were identified and functionally annotated using multiple databases, including VFDB for virulence factors [33], CARD for antibiotic resistance genes [34], and complementary tools such as RAST or Prokka for subsystem classification. The genome sequence has been submitted to the DDBJ under accession number AP044852.

Additionally, predicted protein-coding genes were functionally annotated by assigning them to orthologous groups and KEGG pathways using the KEGG Automatic Annotation Server (KAAS) [35]. Enrichment analysis of core genes within KEGG pathways and Gene Ontology (GO) categories was performed using KOBAS [36]. antiSMASH (v8.0.4) was used to identify biosynthetic gene clusters related to secondary metabolites [37], including those associated with antifungal compounds such as fusaricidin, tridecaptin, and PKS-like products.

### Phylogenetic analysis

To determine the phylogenetic position of *P. polymyxa* CACC1094, two complementary approaches were employed: one based on 16S rRNA gene sequences and the other on whole-genome average nucleotide identity (ANI). For the 16S rRNA gene-based analysis, the sequence of strain CACC1094, together with 24 publicly available *Paenibacillus* sequences retrieved from NCBI, was used to construct a phylogenetic tree. Multiple sequence alignment and tree construction were performed using MEGA X [38] with the Maximum Likelihood method and the Kimura 2-parameter model. Bootstrap analysis was conducted with 1,000 replicates, and branches supported by less than 50% of replicates were collapsed.

Pairwise ANI values were calculated against the whole-genome sequences of 26 *P. polymyxa* strains and two *Paenibacillus sp*. strains using pyani (v0.2.12). ANI values were computed based on MUMmer (ANIm) comparisons, and hierarchical clustering of pairwise similarity matrices was performed to construct a phylogenetic tree. Clustering and dendrogram visualization were generated using the rhierBAPS and dendextend packages in R.

### Genome mining of biosynthetic gene clusters

Secondary metabolite biosynthetic gene clusters (BGCs) in *Paenibacillus polymyxa* CACC1094 were identified using antiSMASH v8.0.4 (https://antismash.secondarymetabolites.org) with default parameters and all extra features enabled [39]. The assembled genome in FASTA format was used as input. Predicted BGCs were classified by biosynthetic type (e.g., NRPS, PKS-like, RiPPs, terpenes), and known clusters were matched against entries in the MIBiG database [40]. Domain architectures of NRPS and PKS clusters were examined using antiSMASH outputs and cross-referenced with Pfam [41] and NCBI CDD [42] databases to confirm the presence of key catalytic and tailoring domains. In addition, homologous clusters were identified using the ClusterBlast function in antiSMASH, which compares query BGCs against the MIBiG reference set and publicly available genomes based on sequence similarity and gene synteny. Representative homologous clusters were visualized for comparison.

### CRISPR-Cas system identification and spacer analysis

CRISPR-Cas loci in *P. polymyxa* CACC1094 were identified from the assembled genome sequence using CRISPRCasTyper [43], which was used to detect CRISPR-Cas loci, annotate associated *cas* genes, and identify CRISPR repeat-spacer arrays. Spacer sequences extracted from all detected arrays were characterized with respect to spacer number, length distribution, and sequence redundancy within and between arrays. Potential protospacer origins were investigated using the standalone command-line version of CRISPRTarget (version CRISPRTarget_cmd_dist.2.2026) [44]. All spacer sequences identified in CACC1094 were queried against plasmid- and phage-associated sequence databases included in the CRISPRTarget package. Detected hits were classified as plasmid- or phage-associated based on database annotations. Spacers returning no significant matches in the searched databases were designated as unmatched.

### Preparation of culture supernatant for metabolite analysis

For seed culture preparation, *Paenibacillus polymyxa* CACC1094 was grown in a 50 mL conical tube containing 20 mL of LB medium at 30°C with shaking at 180 rpm for 24 h. For metabolite analysis, 5% (v/v) of the seed culture was inoculated into a 500 mL Erlenmeyer flask containing 200 mL of sterile Landy medium (glucose, 10.0 g; L-monosodium glutamate, 5.0 g; MgSO_4_, 0.5 g; KCl, 0.78 g; KH_2_PO_4_, 1.0 g; FeSO_4_, 0.05 mg; MnSO_4_, 5.0 mg; CuSO_4_, 0.16 mg; distilled water, 1000 mL; pH 7.2) and incubated at 30°C with shaking at 180 rpm for 48 h. The culture was then centrifuged at 10,000 × g for 20 min at 4°C to remove bacterial cells and debris, and the supernatant was collected. To extract secondary metabolites, the pH of the supernatant was adjusted to below 3.0 by adding 1 N HCl (Sigma-Aldrich) and left at room temperature for 24 h. The resulting precipitate was collected by filtration using a Büchner funnel. The residue retained on the filter was dissolved in methanol (Sigma-Aldrich) and concentrated under a nitrogen stream (T.C.S Pro, Goojung, South Korea) at 35°C for 5 h. The concentrated extract was diluted with 50% methanol prior to LC-QTOF/MS analysis.

### LC-QTOF/MS analysis

Metabolite profiling was performed using an LC-QTOF/MS system (Xevo G2-XS QTOF, Waters, USA) equipped with an ACQUITY Premier BEH C18 column (100 mm × 2.1 mm, 1.7 µm). The column temperature was maintained at 65°C, and the mobile phases consisted of solvent A (water with 0.1% formic acid) and solvent B (acetonitrile with 0.1% formic acid). A linear gradient elution was applied at a flow rate of 0.4 mL min^-1^, with an injection volume of 5 µL.

Mass spectrometric detection was carried out in positive electrospray ionization (ESI⁺) mode over an *m/z* range of 100–1500. The source temperature was set to 150°C, and the capillary voltage was maintained at 3.0 kV. Data acquisition was conducted in Mass Spectrometry Elevated Energy (MSE) mode. The first stage involved a low-collision-energy scan at 6 V, followed by a high-collision-energy scan with collision energies ranging from 20 V to 40 V, using argon as the collision gas.

Data processing and compound annotation were performed using UNIFI software (version 3.8.0.23, Waters). Compound identification was achieved by searching the ChemSpider open library database, and metabolites showing the highest similarity based on fragmentation ion patterns were selected for further analysis.

### RT-PCR and qRT-PCR analyses

For gene expression analysis, the seed culture of *P. polymyxa* CACC1094 was separately inoculated into LB and Landy media and incubated at 30°C with shaking at 180 rpm for 24 h. Bacterial cells grown under each culture condition were harvested for RNA extraction. Total RNA was extracted from CACC1094 cultures using RNeasy Plus Kits (QIAGEN) for RNA Isolation according to the manufacturer’s instructions. First-strand cDNA was synthesized from 1.5 µg of total RNA using M-MLV reverse transcriptase (Promega).

For RT-PCR analysis, cDNA was amplified using gene-specific primers under standard PCR conditions, and the amplification products were analyzed by agarose gel electrophoresis. For quantitative real-time PCR (qRT-PCR), reactions were performed using iTaq Universal SYBR Green Supermix (Bio-Rad) on a CFX96 real-time PCR system (Bio-Rad). Relative gene expression levels were normalized to the 16S rRNA gene and calculated using the comparative cycle threshold (Ct) method. Primer sequences are provided in S2 Table. Three independent biological replicates were analyzed, each with three technical replicates.

### Statistical tests

Quantitative data are expressed as mean ± standard deviation (SD). Inhibition zone measurements were obtained from three independent experiments performed in triplicate; differences among fungal pathogens were assessed by one-way analysis of variance (ANOVA) followed by Tukey’s HSD post hoc test. Relative gene expression data were obtained from three independent replicates; differences in expression between LB and Landy media for each BGC were analyzed using multiple unpaired Student’s *t*-tests with Bonferroni correction for multiple comparisons. Statistical significance was defined as P < 0.05. Specific significance levels and the meaning of symbols (letters and asterisks) are indicated in the respective figure legends. All statistical analyses were performed using JMP16 and GraphPad Prism 11.

## Results

### Isolation and identification of *Paenibacillus polymyxa* CACC1094

A bacterial strain was initially isolated from the bovine rumen contents of a Holstein cow under anaerobic conditions. After isolation, the strain exhibited robust growth under aerobic conditions, forming creamy-white, opaque colonies with irregular margins (S1A Fig), a colony morphology characteristic of the Paenibacillus genus.

For molecular identification, the 16S rRNA gene of the isolate was amplified using universal primers (see Materials and methods) and sequenced. BLASTn analysis of the resulting sequence against the NCBI nucleotide database revealed high similarity to multiple *Paenibacillus polymyxa* strains, showing 100% query coverage and 99.42–99.74% sequence identity (S1B Fig). Based on both colony morphology and 16S rRNA gene similarity, the isolate was identified as *Paenibacillus polymyxa* and hereafter designated as strain CACC1094 (CACC, Cialm Agricultural Cultures Collection).

**Fig 1:**
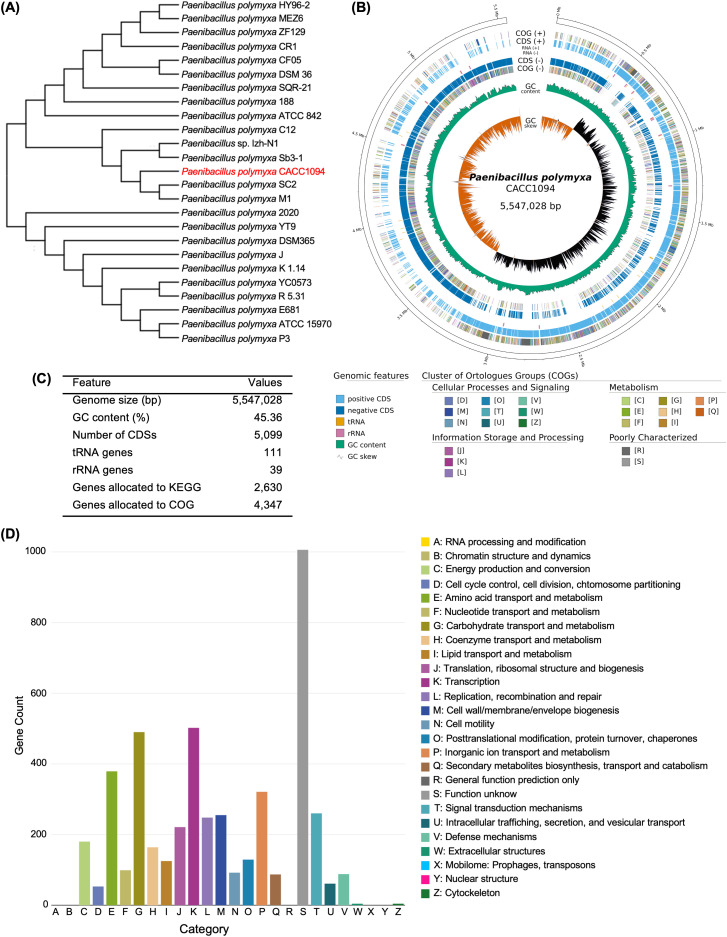
Genomic features, phylogenetic position, and functional classification of *Paenibacillus polymyxa* CACC1094. **(A)** Phylogenetic analysis based on 16S rRNA gene sequences showing the evolutionary relationship of *P. polymyxa* CACC1094 with 24 publicly available *Paenibacillus* strains. The tree was constructed using the Maximum Likelihood method with 1,000 bootstrap replicates, and bootstrap support values ≥50% are shown at branch nodes. Strain CACC1094 is highlighted in red. **(B)** Circular genome map of *P. polymyxa* CACC1094. The outer rings represent coding sequences (CDSs) on the forward (blue) and reverse (light blue) strands. Inner rings indicate positions of tRNA (green), rRNA (red), and genes annotated with COG (Clusters of Orthologous Groups) categories (colored by function). The innermost rings show GC content (black) and GC skew (orange and black). **(C)** Summary of general genome features of *P. polymyxa* CACC1094. **(D)** Functional classification of protein-coding genes based on COG annotations.

To further resolve its taxonomic position, a phylogenetic tree was constructed using 16S rRNA gene sequences from 23 publicly available *P. polymyxa* strains and one *Paenibacillus* sp. strain. The resulting phylogeny placed CACC1094 firmly within the *P. polymyxa* clade, clustering closely with strains Sb3-1, SC2, and M1, indicating a high degree of sequence similarity and shared evolutionary ancestry (Fig 1A). The overall topology also showed clear separation among *P. polymyxa* subgroups, reflecting genetic diversity within the species despite high (>99%) 16S rRNA sequence conservation. Together, these results confirm the taxonomic assignment of CACC1094 to the species *P. polymyxa* and establish it as a member of this phylogenetic group.

### Genome features of CACC1094

To investigate the genomic characteristics of *Paenibacillus polymyxa* CACC1094, whole-genome sequencing and annotation were performed using a hybrid approach that combined Illumina short-read and Oxford Nanopore Technologies (ONT) long-read sequencing. Illumina sequencing generated 4,292,886 reads, yielding approximately 1.27 Gb of data with a GC content of 45.55%. The data quality was high, with 98.29% of bases above Q20 and 88.82% above Q30. ONT sequencing produced 113,903 reads totaling 700,599,121 bp, with a GC content of 45.38%, a read N50 of 9,531 bp, and a mean read quality score of 16.4. The combination of high-accuracy Illumina data and long-range Nanopore reads enabled the construction of a high-quality, contiguous genome assembly.

The assembled genome consisted of a single circular chromosome of 5,547,028 bp with an overall GC content of 45.36%, consistent with other *Paenibacillus* species (Fig 1B and 1C). Genome annotation predicted 5,099 coding DNA sequences (CDSs), 111 tRNA genes, and 39 rRNA genes, indicating a functionally complete genome. Functional categorization revealed that 2,630 genes were assigned to KEGG pathways, while 4,347 genes were grouped into Clusters of Orthologous Groups (COGs), highlighting the strain’s broad metabolic and physiological capabilities (Fig 1C).

The circular genome map illustrates the distribution of CDSs, rRNAs, tRNAs, and COG-annotated genes across the chromosome. Inner tracks display GC content and GC skew, providing insights into genomic architecture and putative replication origin (Fig 1B). BLAST-based taxonomic analysis of each contig obtained from the *de novo* assembly revealed the highest similarity to *Paenibacillus* species, including *Paenibacillus* sp. Izh-N1 (96.04% identity, bitscore 106,700; E-value 0.0) (S1C Fig), confirming the taxonomic affiliation of the genome.

Genome completeness was evaluated using BUSCO (Benchmarking Universal Single-Copy Orthologs) with the *bacillales_odb10* lineage dataset (450 conserved orthologs). A total of 444 BUSCOs (98.7%) were identified as complete, comprising 441 (98.0%) single-copy and 3 (0.7%) duplicated BUSCOs, while 5 (1.1%) were fragmented and only 1 (0.2%) was missing (S2A and S2B Fig). These results confirm that the *P. polymyxa* CACC1094 genome is nearly complete and suitable for downstream functional, comparative, and evolutionary genomic analyses.

### Functional categorization of predicted genes

To gain insights into the functional landscape of the genome, the protein-coding genes of *P. polymyxa* CACC1094 were classified into Clusters of Orthologous Groups (COG). A total of 4,347 genes were assigned to 25 functional categories (Fig 1D). The most abundant category was “S: Function unknown,” comprising more than 1,000 genes, indicating that a substantial proportion of the genome encodes proteins with uncharacterized functions. This was followed by “K: Transcription,” “G: Carbohydrate transport and metabolism,” “E: Amino acid transport and metabolism,” and “P: Inorganic ion transport and metabolism,” reflecting the organism’s broad metabolic versatility and regulatory complexity.

Of particular interest, 87 genes were assigned to “Q: Secondary metabolite biosynthesis, transport, and catabolism,” highlighting the strain’s genomic potential to produce diverse antimicrobial and bioactive compounds. Other notable categories included “T: Signal transduction mechanisms,” “L: Replication, recombination, and repair,” and “M: Cell wall/membrane/envelope biogenesis,” which are likely involved in environmental responsiveness, genome maintenance, and host interaction.

These results collectively underscore the broad functional capacity of *P. polymyxa* CACC1094 and support its potential ecological roles in secondary metabolite production, environmental adaptation, and interactions with plants and other microorganisms.

### Whole-genome phylogeny and ANI-based taxonomic position of CACC1094

**Fig 2:**
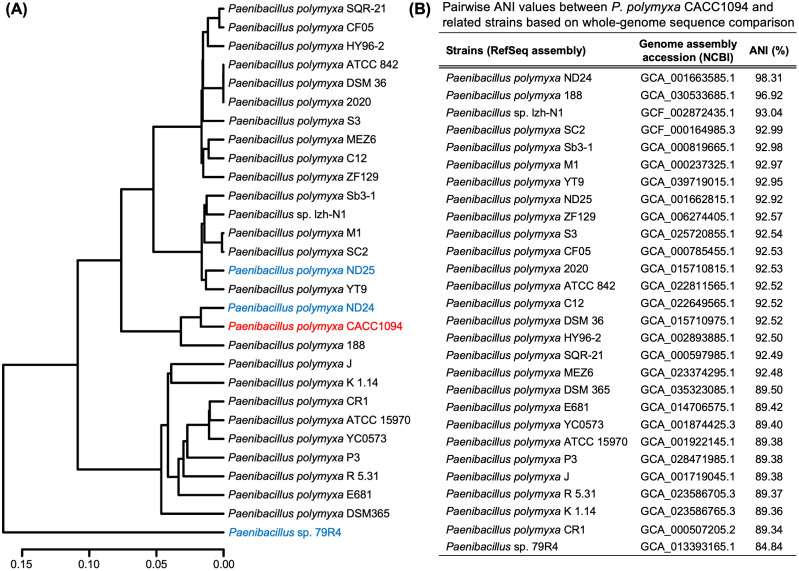
Whole-genome phylogeny and pairwise ANI analysis of *Paenibacillus polymyxa* CACC1094 and related strains. **(A)** Whole-genome phylogenetic tree inferred from concatenated core-genome alignments of *P. polymyxa* CACC1094, 26 publicly available *P. polymyxa* genomes, and two *Paenibacillus* sp. genomes (Izh-N1 and 79R4). *P. polymyxa* CACC1094 is highlighted in red, whereas rumen-associated isolates (ND24, ND25, and 79R4) are highlighted in blue. **(B)** Pairwise average nucleotide identity (ANI) between CACC1094 and the genomes included in panel **(A)**.

Because 16S rRNA gene phylogeny provides limited resolution for distinguishing closely related *Paenibacillus* species, we performed a phylogenomic analysis based on whole-genome sequences. The analysis included 26 publicly available *P. polymyxa* genomes and two *Paenibacillus* sp. genomes. Importantly, the dataset included rumen-associated strains, *P. polymyxa* ND24 and ND25 and *Paenibacillus* sp. 79R4, to enable direct comparison with previously reported bovine rumen isolates (Fig 2A–2B).

As shown in Fig 2A, CACC1094 formed a distinct branch that clustered closely with the rumen-derived *P. polymyxa* ND24 and grouped with P. polymyxa 188, indicating close evolutionary relatedness among these genomes. Pairwise average nucleotide identity (ANI) analysis supported this topology, with CACC1094 sharing 98.31% ANI with ND24 and 96.92% ANI with 188 (Fig 2B). Both values exceed the commonly used species boundary threshold (95–96%), supporting the assignment of CACC1094 to *P. polymyxa*. In contrast, the remaining *P. polymyxa* strains formed three well-supported clusters that were clearly separated from the CACC1094/ND24/188 lineage in the phylogenomic tree (Fig 2A), accompanied by lower ANI values relative to CACC1094 (Fig 2B). Notably, the rumen-derived *P. polymyxa* ND25 showed only 92.92% ANI to CACC1094 and was placed in a distinct subgroup (Fig 2A–2B). Moreover, *Paenibacillus* sp. 79R4, another bovine rumen isolate, exhibited markedly low similarity to CACC1094 (84.84% ANI), indicating that bovine rumen-associated *Paenibacillus* isolates span multiple, taxonomically divergent lineages.

Together, these results indicate that CACC1094 belongs to the *P. polymyxa* species complex and is most closely related to the ND24/188 lineage, while remaining clearly divergent from the ND25-associated subgroup and from distantly related rumen-origin *Paenibacillus* lineages such as 79R4.

### Broad-spectrum antifungal activity of *P. polymyxa* CACC1094

To assess the antifungal spectrum of *P. polymyxa* CACC1094 and link its genomic potential to phenotypic activity, dual-culture assays were conducted against a diverse panel of phytopathogenic fungi, oomycetes, and clinically relevant fungal pathogens. The results revealed broad-spectrum antagonistic activity across multiple tested taxa, underscoring the strain’s biocontrol potential.

**Fig 3:**
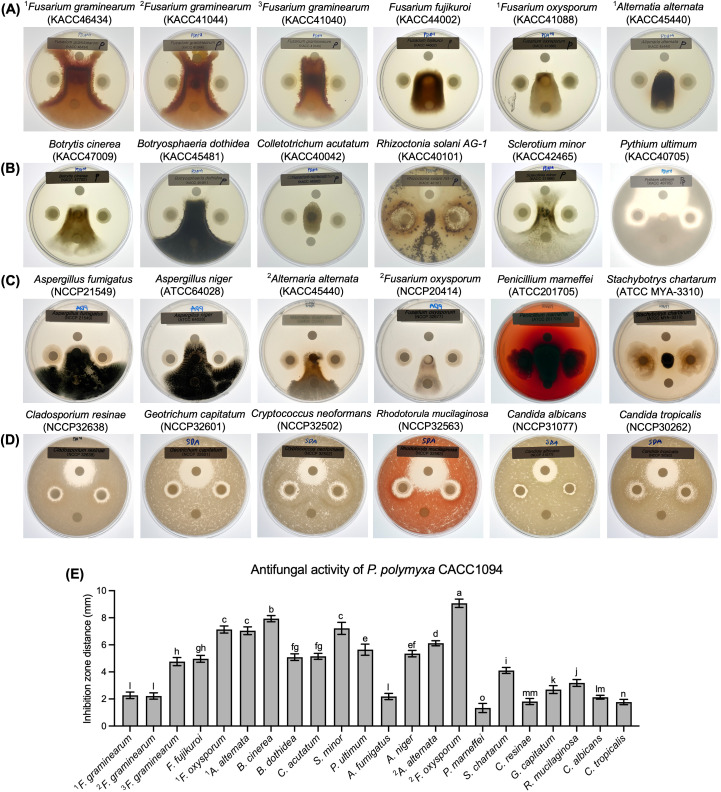
*In vitro* antifungal activity of *Paenibacillus polymyxa* CACC1094 against plant-, animal-, and human-associated fungal pathogens. Dual culture assays were performed to evaluate the antifungal activity of *P. polymyxa* CACC1094 against a panel of fungal and oomycete pathogens. **(A and B)** Plant- pathogenic fungi and oomycetes. **(C and D)** Animal- and human-associated fungal and yeast pathogens. *P. polymyxa* CACC1094 was inoculated onto two paper disks positioned on opposite sides of the plate. One disk containing medium only served as the negative control; the other disk containing hygromycin B (50 µg/µL) served as the positive control. Filamentous fungi and oomycetes were inoculated as 8 mm agar plugs placed at the center of the plate, whereas yeast pathogens were lawn-inoculated onto the agar surface. Plates were incubated for 5 days for filamentous fungi and oomycetes, 2 days for yeast, and 22 days for *Penicillium marneffei* and *Stachybotrys chartarum*. Superscript numerals (e.g., 1, 2, and 3) denote different strains within the same species; corresponding strain accession numbers are provided in parentheses. All assays were performed in triplicate across three independent experiments, and representative images are shown. **(E)** Antifungal activity was quantified as the inhibition zone distance (mm) from the edge of the *P. polymyxa* CACC1094 disk to the nearest point of fungal growth. Data represent mean ± SD from three independent experiments performed in triplicate. Statistical differences were assessed by one-way ANOVA followed by Tukey’s HSD post hoc test; different letters above bars indicate significant differences at *P* < 0.05.

In dual-culture assays against phytopathogenic fungi and oomycetes, *P. polymyxa* CACC1094 exhibited strong growth inhibition across all tested species, including *Fusarium graminearum*, *F. fujikuroi*, *F. oxysporum*, *Alternaria alternata*, *Botrytis cinerea*, *Botryosphaeria dothidea*, *Colletotrichum acutatum*, *Rhizoctonia solani*, *Sclerotium minor*, and *Pythium ultimum* (Fig 3A and 3B). Quantitative analysis of inhibition zones revealed distinct levels of antagonism, ranging from approximately 2–8 mm (Fig 3E). Notably, *B. cinerea*, *A. alternata*, and *S. minor* exhibited strong susceptibility (>7 mm), whereas *F. graminearum* showed moderate inhibition (~2–3 mm) (Fig 3E). In the case of *Rhizoctonia solani* AG-1, no clear inhibition zone was observed, making distance measurement difficult. However, as shown in Fig 3B, dense sporulation occurred toward the negative control but was largely absent near the *P. polymyxa* CACC1094 inoculation site, indicating that CACC1094 effectively suppresses the growth and reproductive activity of *R. solani*.

To investigate cross-domain activity, additional assays were performed against fungal pathogens of clinical and veterinary relevance (Fig 3C and 3D), including *Aspergillus fumigatus*, *A. niger*, *Cladosporium resinae*, *Penicillium marneffei*, *Stachybotrys chartarum*, *Cryptococcus neoformans*, *Candida* spp., and *Malassezia* spp. CACC1094 inhibited the majority of tested pathogens, with *F. oxysporum* showing the largest inhibition (>8 mm), followed by *A. alternata*, *A. niger*, and *S. chartarum* (~4–6 mm) (Fig 3E). In contrast, *A. fumigatus*, *P. marneffei*, and *C. resinae* exhibited minimal inhibition (≤2 mm) (Fig 3E), and *C. glabrata* and *Malassezia* spp. showed no measurable inhibition (≤0.5 mm; S3 Fig).

The observed variation in inhibition profiles likely reflects species-specific resistance mechanisms. It also suggests that multiple bioactive metabolites with distinct modes of action are involved, supporting the potential dual-use application of CACC1094 as a microbial biocontrol agent in both agricultural and veterinary contexts.

### Genome mining identifies diverse biosynthetic gene clusters in *P. polymyxa* CACC1094

**Fig 4:**
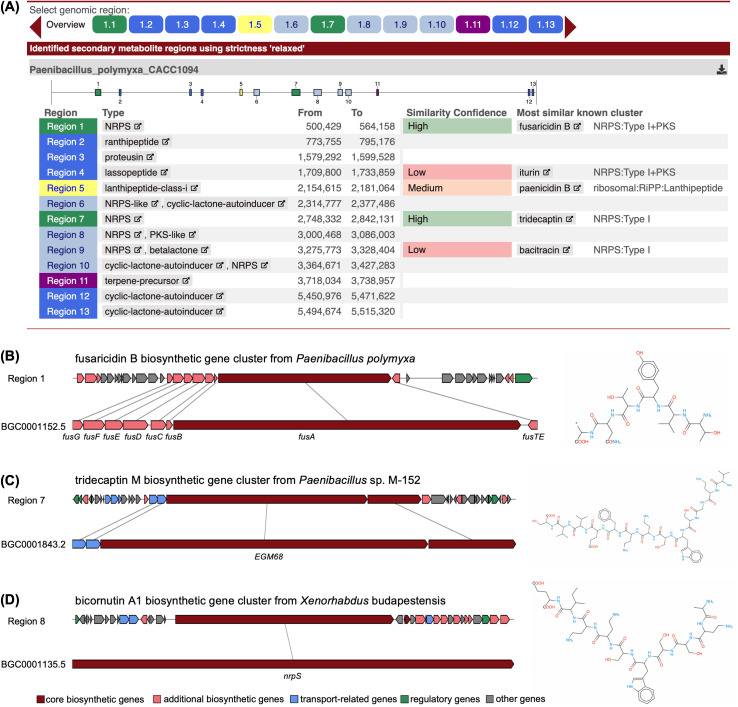
Biosynthetic gene clusters (BGCs) and representative cluster architectures predicted in *Paenibacillus polymyxa* CACC1094. **(A)** Genome mining using antiSMASH 8.0.4 identified secondary metabolite BGCs under relaxed detection stringency. Clusters are categorized by type, genomic location, and similarity confidence level (green: high, orange: medium, red: low). Identified cluster types include non-ribosomal peptide synthetases (NRPS), polyketide synthases (PKS), ribosomally synthesized and post-translationally modified peptides (RiPPs), and other biosynthetic classes. **(B–D)** Representative BGCs and their closest reference clusters with predicted core chemical structures. **(B)** Region 1: fusaricidin B cluster (*P. polymyxa*, BGC0001152.5). **(C)** Region 7: tridecaptin M cluster (*Paenibacillus* sp. M-152, BGC0001843.2). **(D)** Region 8: hybrid NRPS–PKS-like cluster with partial similarity to the bicornutin A1 cluster (*Xenorhabdus budapestensis*, BGC0001135.5). Colored arrows indicate core biosynthetic genes (red), additional biosynthetic genes (pink), regulatory genes (green), transport-related genes (blue), and other genes (gray). Predicted core metabolite structures are shown on the right.

To explore the biosynthetic potential of *Paenibacillus polymyxa* CACC1094, genome mining was performed using antiSMASH version 8.0.4 under relaxed detection stringency. A total of 13 biosynthetic gene clusters (BGCs) were identified, encompassing non-ribosomal peptide synthetase (NRPS), NRPS-like, polyketide synthase (PKS)-like, hybrid NRPS-PKS, and ribosomally synthesized and post-translationally modified peptide (RiPP) clusters, as well as several predicted cyclic-lactone autoinducer and terpene precursor clusters (Fig 4A).

Among the NRPS-associated clusters, Region 1 showed high sequence similarity to the fusaricidin B cluster (NRPS: Type I + PKS class), known for the biosynthesis of antifungal cyclic lipopeptides. Region 7 exhibited strong homology to the tridecaptin cluster, which encodes a linear NRPS product with potent antibacterial activity against Gram-negative bacteria. Regions 4 and 9 displayed low similarity to the iturin and bacitracin clusters, respectively, suggesting the presence of divergent or incomplete BGC variants. The sole RiPP-type cluster, Region 5, was predicted to encode a class I lanthipeptide with moderate similarity to paenicidin B, including a putative precursor peptide gene and associated modification, transport, and immunity genes.

Region 6 was classified as an NRPS-like cluster and was notable for containing cyclic-lactone autoinducer biosynthetic elements, implying a role in microbial communication or metabolite regulation. Region 8 represented a hybrid NRPS–PKS-like cluster with no high-confidence matches to known BGCs, indicating potential for novel metabolite biosynthesis. The remaining BGCs consisted of three cyclic-lactone autoinducer clusters (Regions 10, 12, and 13) and a terpene biosynthetic cluster (Region 11) (Fig 4A). Because of their low similarity to known references, the biological functions of several of these clusters remain uncharacterized.

Collectively, these findings demonstrate that *P. polymyxa* CACC1094 harbors a diverse array of secondary metabolite BGCs, including those encoding well-characterized antimicrobial compounds such as fusaricidin B, tridecaptin, and paenicidin B, as well as multiple low-similarity and hybrid clusters with the potential to produce novel bioactive metabolites.

### In-depth characterization of key antimicrobial BGCs and their predicted products

Building upon the genome mining results, we conducted a detailed comparative analysis of selected BGCs to gain further insights into their structural features and potential bioactivity. Specifically, Regions 1, 7, and 8, representing clusters with high or moderate similarity to known antimicrobial pathways, were analyzed in detail alongside their reference clusters and predicted core structures (Fig 4B–4D).

Region 1 exhibited strong similarity to the *fusaricidin B* cluster from *P. polymyxa*, encoding a hybrid NRPS–PKS pathway that synthesizes cyclic lipopeptides with potent antifungal activity. The predicted core structure contains a conserved cyclic peptide backbone with hydroxylated fatty acid side chains, consistent with known fusaricidin analogs (Fig 4B). Region 7 closely matched the *tridecaptin M* cluster from *Paenibacillus* sp. M-152, a linear NRPS product known for its broad-spectrum antibacterial activity, particularly against Gram-negative bacteria. The core structure prediction revealed a cationic, aromatic-rich peptide consistent with functional tridecaptins (Fig 4C). In contrast, Region 8 represented a hybrid NRPS–PKS-like cluster with only moderate similarity to the *bicornutin A1* cluster from *Xenorhabdus budapestensis*. The predicted peptide exhibits a complex architecture, but lacks a high-confidence match to any known compound, indicating a possible novel metabolite with unexplored bioactivity (Fig 4D).

These findings extend the initial genome mining analysis by linking BGC sequences to putative metabolite structures, thereby emphasizing the chemical diversity and biotechnological potential of *P. polymyxa* CACC1094 as a source of both established and novel antimicrobial agents.

### Transcriptional activity of antimicrobial BGCs under different culture conditions

To investigate whether the identified BGCs are transcriptionally active, we performed RT-PCR and qRT-PCR using BGC-specific primers on *P. polymyxa* CACC1094 cultured in LB and Landy media, the latter of which has been reported to enhance secondary metabolite biosynthesis in *Paenibacillus* species [45,46]. RT-PCR analysis revealed detectable transcription of all examined BGCs (*BGC1*, *BGC3*, *BGC6*, *BGC7*, and *BGC8*) under both culture conditions (S4A Fig). Notably, band intensities for each target were consistently stronger in Landy-grown samples, suggesting higher transcript abundance under this condition. To quantify these differences, qRT-PCR was performed, confirming that transcript levels of all tested *BGCs* were significantly higher in Landy medium than in LB medium (S4B Fig). Among them, *BGC8* showed the greatest induction in Landy medium, while *BGC1* and *BGC7* also exhibited substantial upregulation; *BGC3* and *BGC6* displayed moderate but consistent increases. Collectively, these results indicate that multiple antimicrobial *BGCs* in *P. polymyxa* CACC1094 are transcriptionally active and that their expression is responsive to culture conditions.

### Experimental validation of genome mining predictions by LC-QTOF/MS

**Fig 5:**
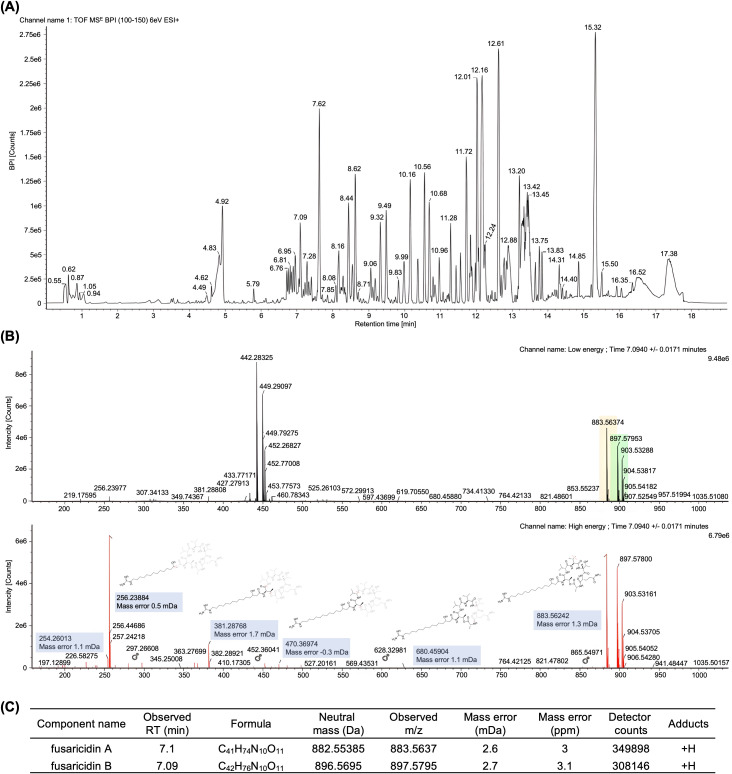
LC-QTOF/MS analysis of fusaricidin-type lipopeptides produced by Paenibacillus polymyxa CACC1094. **(A)** Total ion chromatogram (TIC) of culture supernatant extracted from *P. polymyxa* CACC1094 grown in Landy medium, showing distinct peaks corresponding to secondary metabolites. **(B)** MS/MS fragmentation ion spectra of detected fusaricidin derivatives acquired by LC-QTOF, with diagnostic fragment ions and accurate mass measurements confirming structural identity. **(C)** Summary of detected fusaricidin analogs, including retention time (RT), molecular formula, neutral mass, observed *m*/*z*, mass error, and signal intensity.

Based on the consistently higher transcription of multiple BGCs in Landy medium, we next examined whether Landy-grown cultures produce detectable antimicrobial metabolites. Culture supernatants of *P. polymyxa* CACC1094 grown in Landy medium were analyzed using LC-QTOF/MS to validate the functional expression of the BGCs predicted by genome mining. Distinct chromatographic peaks corresponding to fusaricidin-type lipopeptides were detected at retention times of 7.10 and 7.09 min (Fig 5A). The observed *m*/*z* values of 883.5637 and 897.5795 were in close agreement with the theoretical masses of fusaricidin A (C_41_H_74_N_10_O_11_, 882.5539 Da) and fusaricidin B (C_42_H_76_N_10_O_11_, 896.5695 Da), respectively, with mass errors below 3 ppm (Fig 5B and 5C). MS/MS fragmentation spectra further confirmed the structural identities of these lipopeptides, providing direct experimental evidence that the NRPS cluster predicted by antiSMASH is functionally expressed and produces fusaricidin-type metabolites under laboratory conditions. Taken together, these results provide a direct link between genomic potential and phenotypic antimicrobial activity, demonstrating that *P. polymyxa* CACC1094 not only encodes the biosynthetic capacity for antifungal lipopeptides but also actively produces them *in vitro*.

### Functional genomic features supporting antimicrobial activity and adaptive immunity

Genome-wide annotation of *P. polymyxa* CACC1094 identified multiple genes implicated in antimicrobial metabolite biosynthesis and associated functions (S3 Table). These included NRPS-related genes linked to fusaricidin biosynthesis (*fusA*, *fusAA*, *nrpS1*), a polyketide synthase-related component (*cdaR*), and lanthipeptide biosynthetic enzymes (*nisB*, *nisC*). In addition, genes involved in tailoring, transport, and regulation of secondary metabolites were present, including enzymes associated with amino acid modification and diversification (*ectB*, *gabT*, *aroB*) and regulatory factors such as *abrB*, *spo0A*, *pucR*, and *ndoA*/*ndoA1*. Several additional genes potentially relevant to environmental responses were also annotated (e.g., *yoaJ* and *ispF*) (S3 Table).

*P. polymyxa* CACC1094 also harbors a CRISPR-Cas system comprising both core and accessory components. Identified *cas* genes include components of the adaptation module (*cas1*, *cas2*), an interference-associated subunit (*cas5*), and accessory factors (*csd1*, *csd2*) (S3 Table). The gene rnc, encoding a double-stranded RNA-processing ribonuclease, was identified in proximity to the CRISPR loci. Four CRISPR repeat arrays were detected, each associated with distinct spacer sets.

Spacer analysis of the four arrays revealed 18, 5, 13, and 3 spacers, respectively, totaling 39 spacers. Spacer lengths ranged from 33 to 38 bp, with a median of 35 bp. No identical sequences were detected within or between arrays, indicating that all 39 spacers were non-redundant (S4 Table). Similarity searches identified detectable matches for seven spacers (six to plasmid-associated sequences and one to a phage-associated sequence), with identities ranging from 85.7% to 100%; three spacers showed ≥95% identity to plasmid-derived targets (S4 Table). The remaining 32 spacers had no significant matches in the searched databases.

Together, these findings indicate that CACC1094 encodes genes associated with antimicrobial biosynthesis and CRISPR-Cas-mediated defense, providing genomic context for its antagonistic phenotype.

### Comparative clusterblast analysis of major BGCs in *P. polymyxa* CACC1094

To investigate the evolutionary conservation and distribution of key antimicrobial biosynthetic gene clusters (BGCs) in *P. polymyxa* CACC1094, we performed comparative ClusterBlast analysis using antiSMASH. This approach enabled the alignment of query clusters from CACC1094 with homologous clusters across related *Paenibacillus* species and other bacteria, thereby elucidating their evolutionary conservation, lineage-specific diversification, and potential ecological significance.

ClusterBlast analysis revealed that the fusaricidin B biosynthetic gene cluster was highly conserved across multiple *P. polymyxa* strains, including ZF197, M1, CJX518, YS18-2, ZF129, EB4 G3, SQR-21, S3, and MEZ6. Gene organization and NRPS module composition were nearly identical, indicating strong evolutionary conservation of fusaricidin biosynthesis within the genus. This high degree of conservation underscores the fundamental role of fusaricidin-type lipopeptides in niche competition and microbial antagonism (S5A Fig).

Similarly, the tridecaptin M biosynthetic gene cluster, originally identified in *Paenibacillus* sp. M-152, displayed extensive homology across diverse *P. polymyxa* strains (K16, M1, SC2, C12, 2020, SQR-21, R6.14, A18, MEZ6) as well as *P. terrae* PIC167. While the core NRPS modules were well conserved, minor variation was observed in flanking accessory and tailoring genes. The widespread distribution of this cluster suggests strong selective pressure to maintain antibacterial activity against Gram-negative competitors, highlighting tridecaptin as a common defensive metabolite within the genus (S5B Fig).

In contrast, the bicornutin A1–like biosynthetic gene cluster, originally reported from *Xenorhabdus budapestensis*, exhibited a more heterogeneous distribution and greater structural diversity. Homologous clusters were identified in *P. polymyxa* strains ZF197, CJX518, 2020, M1, SC2, and R6.14, as well as in *Paenibacillus* sp. Izh-N1, *Paenibacillus* sp. M-152, P. peoriae ZBSF16, and even in the distantly related *Clostridium felsineum* DSM 793. Although the core NRPS and trans-AT PKS modules were partially conserved, significant architectural variation, including insertions and rearrangements of accessory genes, was observed. This structural diversity likely reflects adaptive evolution toward niche-specific functions or interactions with distinct microbial communities (S5C Fig).

Overall, comparative ClusterBlast analysis demonstrated that fusaricidin and tridecaptin BGCs are highly conserved antimicrobial traits central to the ecological competitiveness of *P. polymyxa*. In contrast, bicornutin-like clusters showed notable variation in gene organization and accessory gene content across strains, suggesting lineage-specific diversification.

## Discussion

The newly isolated *Paenibacillus polymyxa* CACC1094 exhibited antifungal activity against a broad-spectrum of fungal pathogens. In addition to its strong antagonism toward economically important phytopathogenic fungi and oomycetes, CACC1094 demonstrated clear in vitro inhibitory effects against clinically relevant fungal and yeast species associated with veterinary and human health. This broad-spectrum efficacy highlights the dual potential of *P. polymyxa* CACC1094 as a biocontrol agent for sustainable crop protection and as a valuable source of antifungal metabolites with potential relevance to veterinary applications and future antifungal discovery efforts.

*P. polymyxa* CACC1094 demonstrated potent antifungal activity against a range of phytopathogens, including ascomycetous fungi such as *Fusarium graminearum*, *Fusarium oxysporum*, *Botrytis cinerea*, and *Colletotrichum acutatum*, as well as soil-borne pathogens like *Rhizoctonia solani*, *Sclerotium minor*, and the *oomycete Pythium ultimum*. These pathogens are well known for their environmental persistence and are often difficult to manage with conventional control strategies [1]. Notably, CACC1094 also exhibited strong inhibitory activity against fungal and yeast species of veterinary and clinical relevance, including *Aspergillus* spp., *Alternaria alternata*, *Penicillium marneffei*, *Stachybotrys chartarum*, *Cladosporium resinae*, *Cryptococcus neoformans*, *Geotrichum capitatum*, and *Candida* spp. These species are commonly associated with respiratory or systemic infections, particularly in immunocompromised hosts [47–50]. This dual-spectrum antifungal activity suggests that CACC1094 produces a diverse repertoire of bioactive metabolites effective against phylogenetically distinct fungal taxa. Such versatility enhances its potential as a dual-use biocontrol agent, applicable not only in sustainable agriculture but also as a microbial resource for developing alternative antifungal strategies in veterinary and potentially human medicine, especially in the face of increasing antifungal resistance and limited treatment options.

*Paenibacillus* species are ecologically adaptable and occur in diverse environments, including soil, plant tissues, animal gastrointestinal tracts, and fermented feeds [16]. The bovine rumen, from which CACC1094 was isolated, is a complex anaerobic fermentation chamber where ingested plant material continually introduces soil- and plant-associated bacteria like *Paenibacillus*. These bacteria may act as transient or facultative members of the rumen microbiome [51,52]. In this environment, such bacteria can contribute to fiber degradation, the production of volatile fatty acids (VFAs), and the suppression of undesirable microbes through the production of antimicrobial metabolites. Given this ecological context, rumen-derived *P. polymyxa* strains may possess biosynthetic repertoires shaped by both their plant-associated origins and their adaptation to the competitive, microbially dense rumen environment. These dual ecological pressures likely enhance their capacity to produce a broader range of secondary metabolites, which may explain the observed antifungal activity against both plant pathogens and animal and human fungal pathogens.

Although CACC1094 was isolated from bovine rumen, isolation alone does not establish whether the strain actively proliferates as a persistent rumen member or represents a transient organism introduced with feed. The rumen is continuously inoculated by microorganisms carried in with feed and water, and many facultative bacteria detected in the rumen are considered nonindigenous and transient populations [53]. In addition, rumen digesta is subject to ongoing passage and washout dynamics, meaning that microorganisms may be detected even if they do not maintain sustained growth in situ [53,54]. In this context, a feed-origin scenario is biologically plausible because *Paenibacillus* species have been isolated directly from animal feed, supporting the possibility that spore-forming *Paenibacillus* can enter the rumen via dietary inputs and be recovered without long-term colonization [55]. At the same time, evidence from cultured rumen isolates suggests that certain *Paenibacillus* lineages can persist and function under rumen-relevant conditions. For example, a denitrifying *Paenibacillus* strain (79R4) was isolated from the bovine rumen and selected for enhanced nitrite-reducing activity [23], indicating that at least some rumen-derived *Paenibacillus* strains can express traits consistent with rumen metabolism and competition. Accordingly, the genomic features of CACC1094 (e.g., antimicrobial BGC repertoire and defense systems) may be interpreted as traits that could provide a competitive advantage in a dense microbial ecosystem; however, we conservatively describe CACC1094 as rumen-associated because direct evidence of active growth in the rumen is currently unavailable. Future work combining longitudinal sampling, strain-targeted quantification, and anaerobic growth/fermentation assays using rumen-relevant substrates would clarify whether CACC1094 actively proliferates in the rumen or represents dietary transit.

Phylogenomic analysis provided important context for interpreting the genomic and functional characteristics of CACC1094. In whole-genome comparisons including 26 publicly available *P. polymyxa* genomes and *Paenibacillus* sp. genomes, CACC1094 was positioned within the *P. polymyxa* species complex and clustered most closely with the rumen-derived strain ND24 and *P. polymyxa* strain 188. Consistent with this topology, CACC1094 shared 98.31% ANI with ND24 and 96.92% ANI with 188, whereas ANI values with all other strains were below 93.04%. This finding places CACC1094 firmly within the *P. polymyxa* species boundary while highlighting its marked genomic divergence from other known isolates. The distinct phylogenetic position of CACC1094, forming a separate branch with ND24/188 strains rather than clustering with other *P. polymyxa* subgroups, suggests the presence of an evolutionarily divergent lineage. Such genomic distinctiveness may underlie its unique biosynthetic repertoire and unusually broad antifungal activity profile.

Whole-genome sequencing of *P. polymyxa* CACC1094 provided insights into the biosynthetic potential underlying its broad-spectrum antifungal activity. The assembled genome spans 5.55 Mb with a GC content of 45.36%, comprising 5,099 predicted coding sequences (CDSs). This genomic architecture is consistent with those of other well-characterized *P. polymyxa* strains, including E681, SC2, 188, WLY78, and WR-2 [20,56–59]. Although these reference strains are recognized for their antifungal capabilities, CACC1094 demonstrated a broader antagonistic spectrum *in vitro*, inhibiting not only a wide range of economically significant phytopathogens but also fungal and yeast species associated with veterinary and human diseases. This extended activity profile suggests that CACC1094 may harbor distinct or more diversely composed BGCs that contribute to its antifungal efficacy across a phylogenetically broad range of fungal taxa.

Notably, antiSMASH analysis of the CACC1094 genome revealed multiple biosynthetic gene clusters (BGCs) associated with antimicrobial metabolite production, including nonribosomal peptide synthetase (NRPS) clusters predicted to encode fusaricidin and tridecaptin, a lanthipeptide-class-I cluster potentially encoding paenicidin, and additional uncharacterized clusters such as a polyketide synthase (PKS)-like BGC. Among these, NRPS-derived metabolites like fusaricidin and tridecaptin have been relatively well characterized for their antimicrobial properties, whereas the contribution of PKS, hybrid NRPS-PKS, or lanthipeptide clusters to antifungal activity remains less clearly defined and warrants further investigation [18–20,59–61].

Comparative genomic analyses across *P. polymyxa* strains have shown that most antimicrobial BGCs are derived from NRPS pathways, which typically encode lipopeptides such as polymyxins, fusaricidins, and tridecaptins [16,22,59,62–64]. Among these, fusaricidin is particularly notable for its potent antifungal activity. First described by Kajimura and Kaneda [64], fusaricidin is a cyclic hexapeptide linked to a long-chain fatty acid that facilitates membrane insertion and pore formation, ultimately causing cytoplasmic leakage and fungal cell death [63,64]. The presence of a fusaricidin BGC in CACC1094 supports the hypothesis that membrane-targeting lipopeptides play a central role in its antifungal mechanism. This is further corroborated by the observed inhibition of *Pythium ultimum*, *Rhizoctonia solani*, and *Fusarium* spp., all of which are known to be susceptible to fusaricidin-like compounds [65].

Tridecaptins were also identified in the CACC1094 genome. Originally characterized for their antibacterial activity, particularly through disruption of the proton motive force in Gram-negative bacteria [66,67], tridecaptins have more recently been implicated in broader microbial interactions. These may include modulation of rhizosphere microbial communities, interference with biofilm formation, or competitive exclusion, all of which could indirectly contribute to fungal suppression. The co-expression of tridecaptins with fusaricidins in CACC1094 raises the possibility of synergistic mechanisms that enhance its antagonistic efficacy. While the direct antifungal role of tridecaptins remains to be elucidated, their presence in a strain exhibiting broad-spectrum antifungal activity warrants further functional investigation.

In addition to these NRPS-associated clusters, a lanthipeptide-class-I BGC potentially encoding paenicidin was identified. Lanthipeptides, such as nisin and paenicidin, are ribosomally synthesized and post-translationally modified peptides best known for their potent antibacterial activity against Gram-positive bacteria through lipid II binding and pore formation [68,69]. Although their direct contribution to antifungal activity remains unclear, their presence highlights the biosynthetic versatility of CACC1094 and suggests potential dual functionality as both an antibacterial and antifungal resource. Moreover, given the growing recognition that bacterial secondary metabolites can influence microbial community dynamics, it is possible that lanthipeptides contribute indirectly to fungal suppression by shaping competitive interactions within the rhizosphere or host-associated microbiomes.

Interestingly, while polymyxins have historically been regarded as hallmark metabolites of *P. polymyxa*, no polymyxin BGCs were detected in the genome of CACC1094. This absence suggests that its antifungal activity arises primarily from alternative NRPS pathways, namely fusaricidin and tridecaptin, and potentially from other novel metabolites.

Finally, the PKS-like cluster detected in CACC1094 adds an intriguing dimension to its biosynthetic potential. Polyketides are structurally diverse natural products with a broad range of biological activities, including antifungal, antibacterial, and anticancer properties [70]. The PKS-like cluster identified in CACC1094 exhibited low sequence similarity to known BGCs in the MIBiG database, strongly suggesting the potential for the biosynthesis of a previously uncharacterized polyketide compound. Although the function of this cluster remains unverified, its uniqueness reinforces the untapped metabolic diversity of *P. polymyxa* and highlights the utility of genome-guided discovery in identifying novel biocontrol agents. Future work involving LC-MS/MS profiling, gene knockout studies, and heterologous expression will be essential for determining its structure, mode of action, and contribution to the strain’s overall antifungal activity.

Collectively, these genomic findings strongly support the notion that CACC1094 synthesizes a diverse repertoire of secondary metabolites that underlie its broad antifungal efficacy. The detection of conserved BGCs for fusaricidin and tridecaptin, in conjunction with a novel PKS-like cluster, provides molecular evidence for its inhibitory effects against both plant- and animal-associated fungal pathogens. Nevertheless, the translation of these genomic capacities into actual antifungal outcomes revealed clear species-specific differences, emphasizing the need to integrate genome mining with susceptibility testing.

The coexistence of antimicrobial biosynthetic genes and CRISPR-Cas immunity components in *P. polymyxa* CACC1094 reflects a dual functional capacity: the production of diverse bioactive metabolites alongside defense against foreign genetic elements. This combination may contribute to the stability of biosynthetic gene clusters by limiting disruptive horizontal gene transfer events, potentially conferring a competitive advantage in complex microbial environments. This functional duality may reflect selective pressures encountered in both plant-associated and rumen ecosystems, although direct experimental evidence for this inference remains to be established.

Supporting this interpretation, the CRISPR spacer inventory provides more direct evidence for the adaptive immunity profile of CACC1094. Four arrays comprising 39 non-redundant spacers are consistent with repeated historical exposure to diverse mobile genetic elements. Of these, only seven spacers showed detectable similarity to sequences in public databases, with matches predominantly to plasmid-associated targets and one phage-associated target. The limited number of database matches likely reflects the underrepresentation of host- and rumen-associated mobilomes in current sequence collections rather than a restricted exposure history, suggesting that the full scope of mobile genetic elements encountered by CACC1094 remains incompletely captured by available reference data. A systematic comparison of spacer repertoires across *P. polymyxa* genomes would provide valuable evolutionary context, for example, whether the plasmid-biased targeting observed in CACC1094 is a lineage-specific trait or broadly conserved within the species. This remains an important direction for future investigation.

Of note, *P. polymyxa* CACC1094 exhibited clear antifungal activity against *Candida albicans* and *C. tropicalis*, but not against *C. glabrata*, highlighting the importance of species-specific physiological traits in determining susceptibility. The reduced sensitivity of *C. glabrata* likely stems from its distinct evolutionary lineage, closer to *Saccharomyces cerevisiae* than to *C. albicans*, and the absence of key virulence factors such as hyphal formation and secreted proteases. Instead, *C. glabrata* exhibits exceptional stress tolerance, a rigid and compact cell wall, and upregulated efflux pumps, all contributing to its intrinsic resistance to antifungal agents, including lipopeptides like fusaricidin and tridecaptin [71–73]. These observations align with clinical evidence indicating the need for alternative antifungal therapies for *C. glabrata* infections due to reduced susceptibility to azoles and other commonly used agents [49]. In contrast, *C. albicans* and *C. tropicalis* possess more permeable cell walls enriched in *β*-glucans and mannans, facilitating interaction with amphiphilic secondary metabolites [74]. This structural difference may underlie their increased susceptibility to CACC1094-derived lipopeptides, underscoring the need for strain-specific susceptibility testing when developing microbial biocontrol agents.

Furthermore, CACC1094 showed no antifungal activity against *Malassezia pachydermatis* and *M. furfur*, lipid-dependent yeasts commonly associated with dermatological conditions in animals and humans. This resistance is likely attributable to the unique cell wall composition and lipid metabolism of *Malassezia*. These species lack fatty acid synthase genes and depend on external lipids, resulting in a highly hydrophobic surface [75,76]. Additionally, their cell walls are enriched with chitin, melanin-like pigments, and atypical β-glucan branching, which may hinder the penetration of membrane-active lipopeptides [76,77]. Intrinsic resistance mechanisms and oxidative stress tolerance further reduce their susceptibility [78]. These findings emphasize the need for targeted screening strategies against lipid-dependent fungi when evaluating bacterial biocontrol strains.

From an application perspective, *P. polymyxa* CACC1094 demonstrates strong potential in both agricultural and health-related contexts. In crop systems, the strain inhibited key pathogens of cereals (*F. graminearum*, *F. oxysporum*, *R. solani*), vegetables (*B. cinerea*, *S. minor*), and fruit trees (*A. alternata*, *B. dothidea*, *C. acutatum*), suggesting broad utility across diverse cropping systems. Practical deployment could include seed treatments, soil inoculants, or root drench formulations, which would enable efficient rhizosphere colonization. Genomic features further indicate a capacity for stable metabolite production under variable environmental conditions, a critical trait for field robustness. Moreover, its integration into microbial consortia is particularly promising. Multi-strain consortia with complementary biosynthetic repertoires often outperform single-strain applications in terms of efficacy and environmental stability [79]. Given that CACC1094 harbors at least three distinct biosynthetic gene clusters, it could serve as a keystone strain within such synthetic microbial communities.

Beyond agriculture, CACC1094 exhibited potent antifungal activity against clinically and veterinary relevant fungi and yeasts, including *Aspergillus* spp., *Alternaria alternata*, *Penicillium marneffei*, *Stachybotrys chartarum*, *Cladosporium resinae*, *Cryptococcus neoformans*, *Geotrichum capitatum*, *Rhodotorula mucilaginosa*, and *Candida* spp. These pathogens are associated with respiratory, systemic, or opportunistic infections in both humans and animals and often exhibit intrinsic or acquired resistance to conventional antifungals [80,81]. These findings suggest that CACC1094 may have potential beyond agricultural biocontrol, warranting further investigation into its applicability in veterinary and clinical contexts. Importantly, the strain exhibited selective antifungal activity, strongly inhibiting *Candida albicans* and *C. tropicalis* but not *C. glabrata* or *Malassezia* spp. This selective profile underscores the need for comprehensive, species-specific susceptibility testing when considering therapeutic applications beyond agriculture. Further in vivo validation, particularly in veterinary clinical models, will be essential to confirm its safety, efficacy, and practical utility in animal health.

In terms of novelty, while numerous *P. polymyxa* strains have been sequenced and tested for antimicrobial properties, relatively few have been characterized through an integrated approach that combines phenotypic screening with genome mining. Our findings address this gap and lay the groundwork for further exploration. Future studies should aim to (i) isolate and structurally characterize the active metabolites produced by CACC1094, (ii) evaluate in planta efficacy using economically important crops, (iii) assess rhizosphere colonization dynamics under field-like conditions, and (iv) perform thorough safety and toxicity assessments to meet regulatory standards for both agricultural and veterinary applications.

Altogether, these results position *P. polymyxa* CACC1094 as a promising dual-use biocontrol agent. Its demonstrated broad-spectrum activity, coupled with a well-defined genomic basis for secondary metabolite production, supports its potential to address pressing challenges in sustainable agriculture and animal health. Further translational efforts will be key to advancing this rumen-associated *P. polymyxa* isolate from laboratory discovery to field and clinical implementation.

## Conclusion

This study reports the isolation and genome-guided characterization of *Paenibacillus polymyxa* CACC1094 from the bovine rumen, demonstrating broad-spectrum antifungal activity spanning plant-pathogenic, veterinary, and clinically relevant fungal taxa. Dual-culture assays revealed potent antagonism against economically important phytopathogens (*Fusarium* spp., *Botrytis cinerea*, *Pythium ultimum*, and others) as well as opportunistic fungal pathogens of veterinary and clinical relevance (*Aspergillus* spp., *Cryptococcus neoformans*, and *Candida* spp.), underscoring the strain’s broad cross-kingdom inhibitory range.

Whole-genome sequencing and antiSMASH-based genome mining revealed 13 BGCs, including those encoding fusaricidin, tridecaptin, and a lanthipeptide-type paenicidin-like cluster. Notably, the identification of a hybrid NRPS–PKS cluster lacking close reference counterparts underscores the strain’s untapped biosynthetic potential and opens avenues for the discovery of structurally novel bioactive compounds. LC-QTOF/MS analysis confirmed the production of fusaricidin A and fusaricidin B under laboratory conditions, establishing a direct link between genomic potential and actual metabolite production. Transcriptomic profiling further demonstrated that multiple BGCs are transcriptionally active and differentially upregulated under secondary metabolite-inducing conditions, reinforcing their functional relevance.

Phylogenomic analysis placed CACC1094 within a distinct sublineage of the *P. polymyxa* species complex, most closely related to the rumen-derived strain ND24 (ANI: 98.31%) and strain 188 (ANI: 96.92%), while remaining clearly divergent from all other examined strains (ANI: ≤ 93%). This genomic distinctiveness, coupled with the presence of a complete CRISPR-Cas adaptive immune system, suggests that CACC1094 integrates antimicrobial biosynthesis with defense against mobile genetic elements, a dual strategy likely shaped by the competitive microbial ecology of the rumen. However, the precise ecological drivers of this adaptation remain to be experimentally validated.

Collectively, these results establish *P. polymyxa* CACC1094 as a promising dual-use biocontrol candidate, whose broad antagonistic spectrum is supported by a well-characterized genomic basis for secondary metabolite production. To advance this strain toward practical application, future research should prioritize: (i) the isolation and structural characterization of active metabolites beyond fusaricidins, particularly the uncharacterized hybrid NRPS–PKS product; (ii) in planta and *in vivo* efficacy evaluations in relevant agricultural and veterinary models; (iii) the assessment of rhizosphere colonization dynamics and environmental persistence; and (iv) comprehensive biosafety and regulatory evaluations for both sectors. The bovine rumen represents an underexplored reservoir of biosynthetically diverse *Paenibacillus* lineages, and this study provides a foundation for their systematic utilization as next-generation biocontrol agents.

## Supporting information

S1 Fig Taxonomic identification of *Paenibacillus polymyxa* CACC1094. **(A)** Colony morphology of strain CACC1094 grown on LB agar. **(B)** BLASTn analysis of the 16S rRNA gene sequence showing high similarity to multiple *P. polymyxa* reference strains. **(C)** Representative contig-level BLAST analysis indicating the closest match to *Paenibacillus* sp. Izh-N1.
(TIFF)

S2 Fig Genome completeness of *Paenibacillus polymyxa* CACC1094 assessed using BUSCO. **(A)** Summary bar chart of BUSCO assessment results based on the bacillales_odb10 lineage dataset (*n* = 450). **(B)** Tabular summary of BUSCO statistics, including the number and proportion of complete (single-copy and duplicated), fragmented, and missing BUSCOs.
(TIFF)

S3 Fig Lack of antagonistic activity of *Paenibacillus polymyxa* CACC1094 against resistant yeasts. Dual culture assays showed no growth inhibition of the lipid-dependent yeasts *Malassezia pachydermatis* (KCTC27587) and *M. furfur* (KCTC7546), as well as the intrinsically resistant yeast *Candida glabrata* (NCCP30399). *P. polymyxa* CACC1094 was inoculated onto two side-positioned paper disks (left and right), with the lower disk serving as a negative control (medium only) and the upper disk containing a positive control (hygromycin B, 50 µg/µL). Yeast pathogens were spread evenly across the surface of the plate. Plates were incubated for 2 days. Assays were performed in triplicate and repeated twice independently. Representative images are shown.
(TIFF)

S4 Fig Transcriptional activity of selected *biosynthetic gene clusters* (*BGCs*) in *Paenibacillus polymyxa* CACC1094 under different culture conditions. **(A)** RT-PCR analysis of *BGC* expression in *P. polymyxa* CACC1094 grown in LB and Landy media. Total RNA was extracted from cultured cells, reverse-transcribed into cDNA, and amplified using *BGC*-specific primer sets targeting *BGC1*, *BGC3*, *BGC6*, *BGC7*, and *BGC8*. The 16S rRNA gene was used as an internal control. **(B)** Quantitative real-time PCR (qRT-PCR) analysis of relative transcript levels of *BGC1*, *BGC3*, *BGC6*, *BGC7*, and *BGC8* in *P. polymyxa* CACC1094 grown in LB and Landy media. Relative expression levels were calculated using the comparative cycle threshold method with 16S rRNA as the reference gene. Statistical significance between LB and Landy for each *BGC* was assessed using multiple unpaired Student’s *t* tests with Bonferroni correction. Data are presented as mean ± SD from three independent replicates (*n* = 3). ****, *P* < 0.0001.
(TIFF)

S5 Fig ClusterBlast analysis of major biosynthetic gene clusters identified in *Paenibacillus polymyxa* CACC1094. **(A)** *Fusaricidin B* biosynthetic gene cluster, **(B)** *Tridecaptin M* biosynthetic gene cluster, and **(C)** *Bicornutin A1–like* biosynthetic gene cluster. The query clusters were compared against publicly available genomes using antiSMASH ClusterBlast, which identifies homologous gene clusters based on sequence similarity and synteny.
(TIFF)

S1 Table List of fungal and yeast pathogens, including crop-associated, respiratory, opportunistic, and veterinary dermatological species, used for antifungal activity assays of *Paenibacillus polymyxa* CACC1094. (XLSX)

S2 Table Gene-specific primer sequences used for RT-PCR and qRT-PCR analyses. (XLSX)

S3 Table Antimicrobial activity-related and CRISPR-associated genes identified in the genome of *Paenibacillus polymyxa* CACC1094. (XLSX)

S4 Table CRISPR spacer inventory and putative protospacer matches of *Paenibacillus polymyxa* CACC1094. (XLSX)
