# Supplementary figures and images for: Broad-spectrum antifungal activity and genome-guided characterization of Paenibacillus polymyxa CACC1094 isolated from the bovine rumen
Source: PLoS One. 2026 Jun 25;21(6):e0350885. doi: 10.1371/journal.pone.0350885 (PMC13298791; doi:10.1371/journal.pone.0350885)

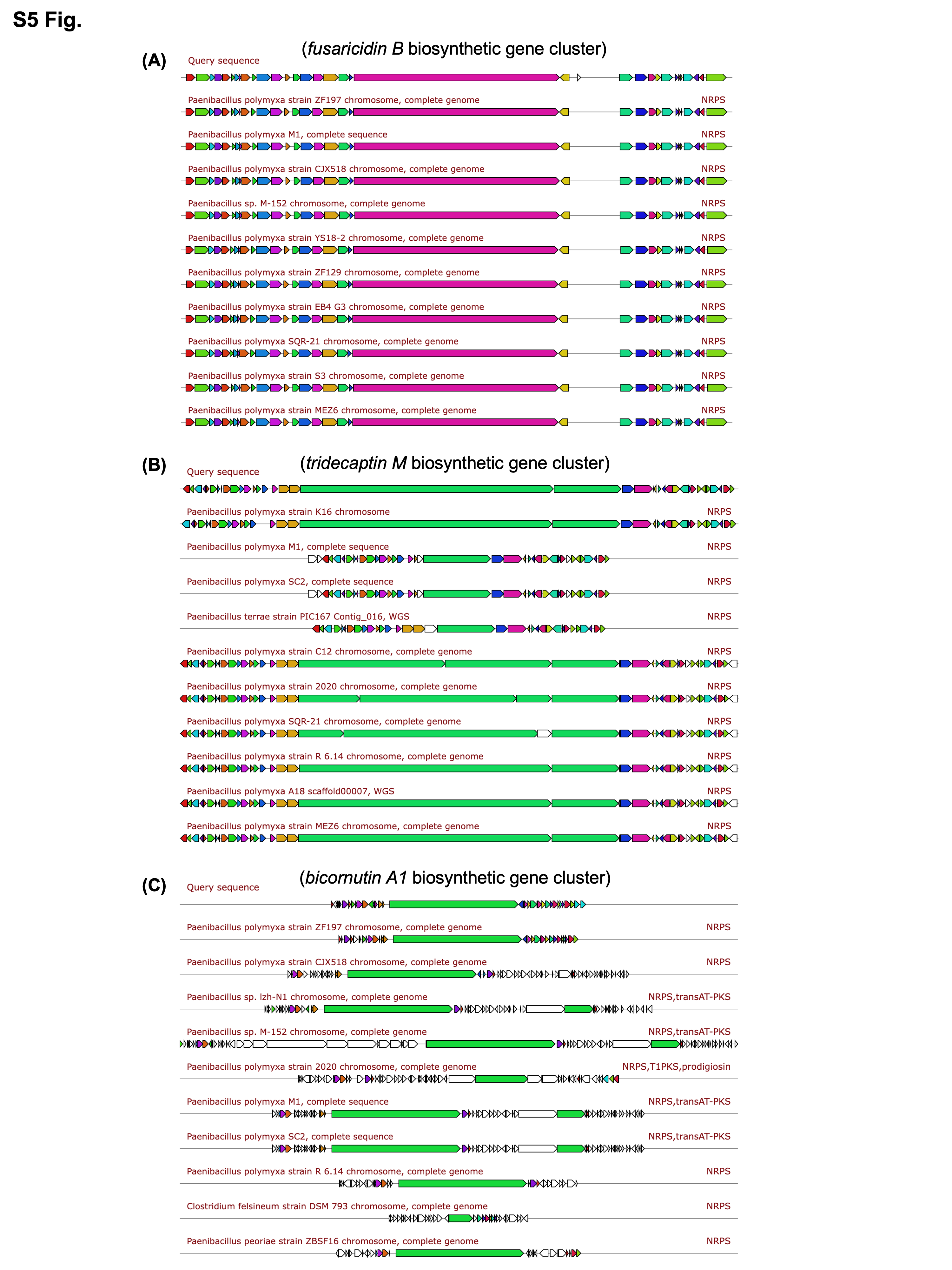

Supplement: S1 Fig — (A) Colony morphology of strain CACC1094 grown on LB agar. (B) BLASTn analysis of the 16S rRNA gene sequence showing high similarity to multiple P. polymyxa reference strains. (C) Representative contig-level BLAST analysis indicating the closest match to Paenibacillus sp. Izh-N1. (TIFF) [file pone.0350885.s001.tiff]

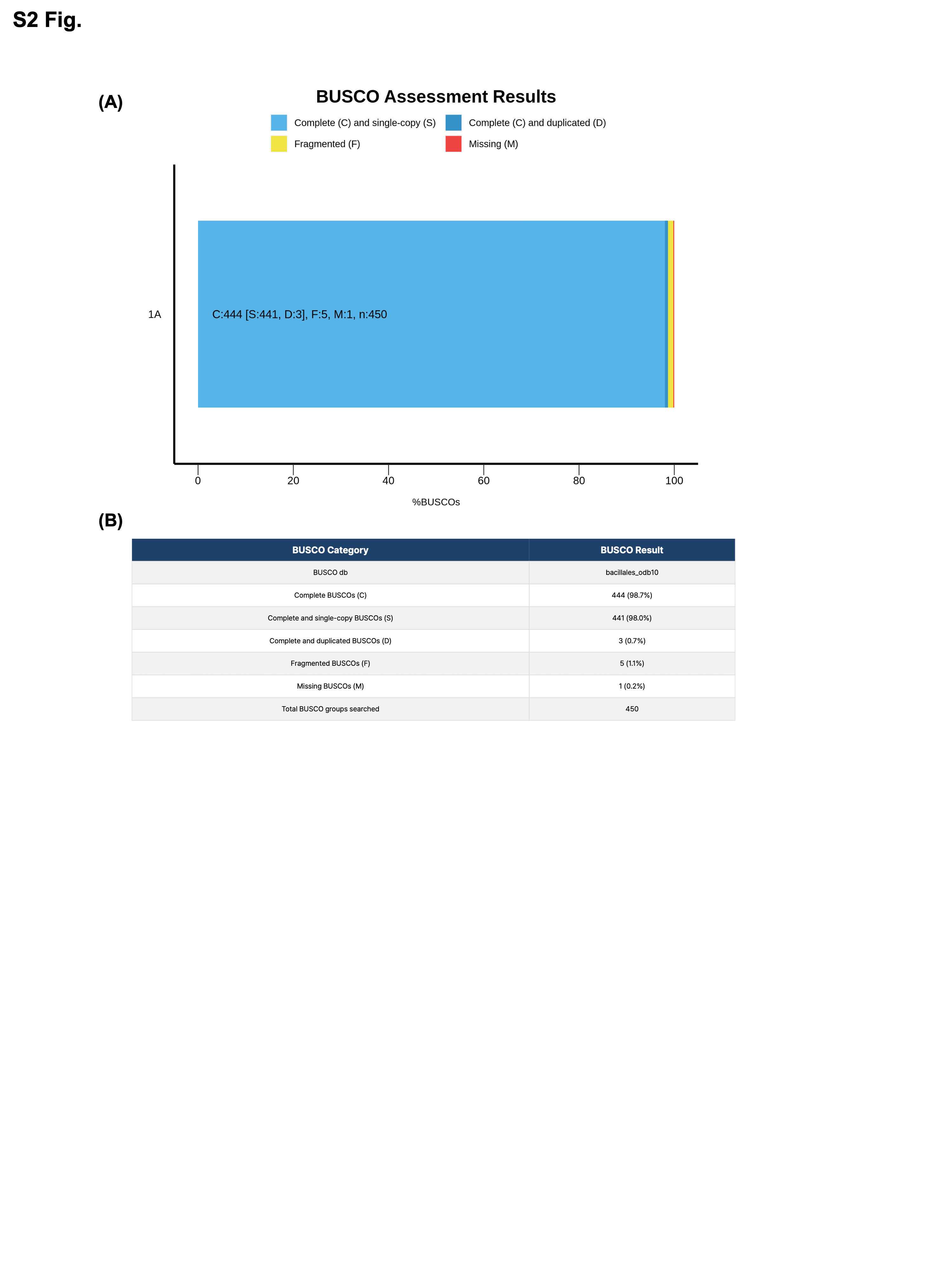

Supplement: S2 Fig — (A) Summary bar chart of BUSCO assessment results based on the bacillales_odb10 lineage dataset (n = 450). (B) Tabular summary of BUSCO statistics, including the number and proportion of complete (single-copy and duplicated), fragmented, and missing BUSCOs. (TIFF) [file pone.0350885.s002.tiff]

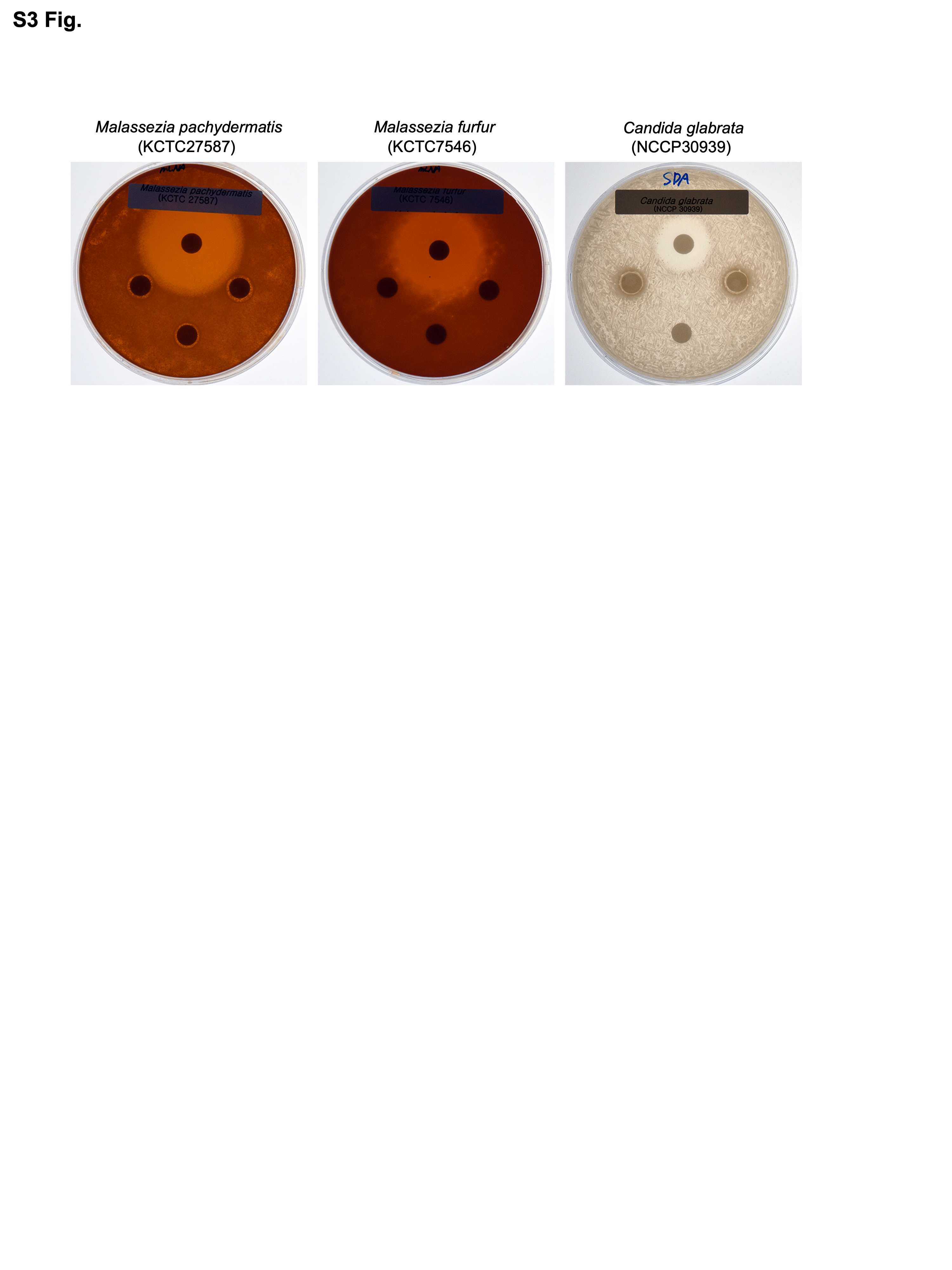

Supplement: S3 Fig — Dual culture assays showed no growth inhibition of the lipid-dependent yeasts Malassezia pachydermatis (KCTC27587) and M. furfur (KCTC7546), as well as the intrinsically resistant yeast Candida glabrata (NCCP30399). P. polymyxa CACC1094 was inoculated onto two side-positioned paper disks (left and right), with the lower disk serving as a negative control (medium only) and the upper disk containing a positive control (hygromycin B, 50 µg/µL). Yeast pathogens were spread evenly across the surface of the plate. Plates were incubated for 2 days. Assays were performed in triplicate and repeated twice independently. Representative images are shown. (TIFF) [file pone.0350885.s003.tiff]

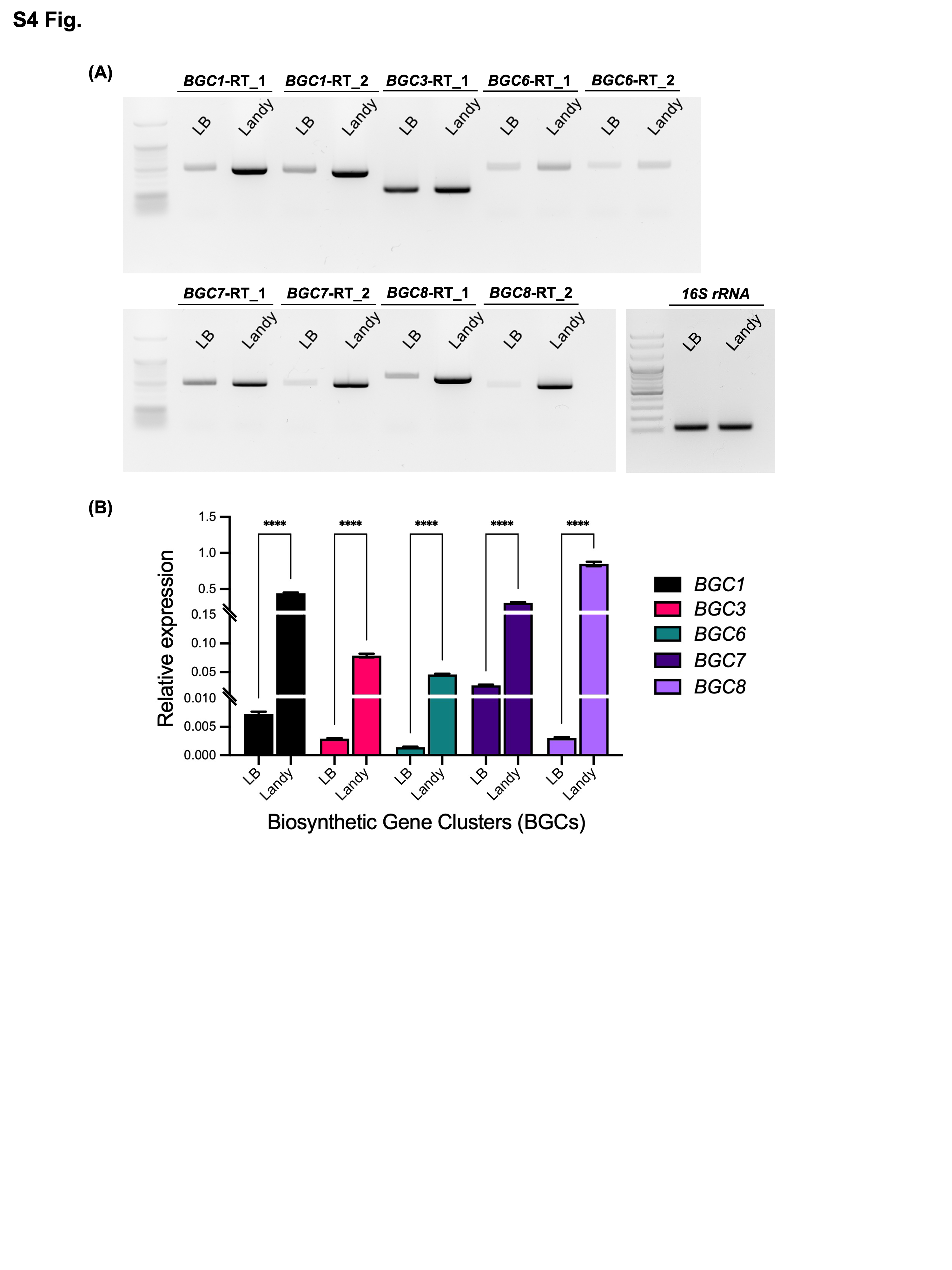

Supplement: S4 Fig — (A) RT-PCR analysis of BGC expression in P. polymyxa CACC1094 grown in LB and Landy media. Total RNA was extracted from cultured cells, reverse-transcribed into cDNA, and amplified using BGC-specific primer sets targeting BGC1, BGC3, BGC6, BGC7, and BGC8. The 16S rRNA gene was used as an internal control. (B) Quantitative real-time PCR (qRT-PCR) analysis of relative transcript levels of BGC1, BGC3, BGC6, BGC7, and BGC8 in P. polymyxa CACC1094 grown in LB and Landy media. Relative expression levels were calculated using the comparative cycle threshold method with 16S rRNA as the reference gene. Statistical significance between LB and Landy for each BGC was assessed using multiple unpaired Student’s t tests with Bonferroni correction. Data are presented as mean ± SD from three independent replicates (n = 3). ****, P < 0.0001. (TIFF) [file pone.0350885.s004.tiff]
